# Engineered Microgels—Their Manufacturing and Biomedical Applications

**DOI:** 10.3390/mi12010045

**Published:** 2021-01-01

**Authors:** Hamzah Alzanbaki, Manola Moretti, Charlotte A. E. Hauser

**Affiliations:** Laboratory for Nanomedicine, Division of Biological and Environmental Science and Engineering, King Abdullah University of Science and Technology, 4700 Thuwal, Jeddah 23955-6900, Saudi Arabia; hamzah.alzanbaki@kaust.edu.sa (H.A.); manola.moretti@kaust.edu.sa (M.M.)

**Keywords:** microgels, self-assembling peptides, biofabrication, 3D bioprinting, cell-laden constructs

## Abstract

Microgels are hydrogel particles with diameters in the micrometer scale that can be fabricated in different shapes and sizes. Microgels are increasingly used for biomedical applications and for biofabrication due to their interesting features, such as injectability, modularity, porosity and tunability in respect to size, shape and mechanical properties. Fabrication methods of microgels are divided into two categories, following a top-down or bottom-up approach. Each approach has its own advantages and disadvantages and requires certain sets of materials and equipments. In this review, we discuss fabrication methods of both top-down and bottom-up approaches and point to their advantages as well as their limitations, with more focus on the bottom-up approaches. In addition, the use of microgels for a variety of biomedical applications will be discussed, including microgels for the delivery of therapeutic agents and microgels as cell carriers for the fabrication of 3D bioprinted cell-laden constructs. Microgels made from well-defined synthetic materials with a focus on rationally designed ultrashort peptides are also discussed, because they have been demonstrated to serve as an attractive alternative to much less defined naturally derived materials. Here, we will emphasize the potential and properties of ultrashort self-assembling peptides related to microgels.

## 1. Introduction

Hydrogels are a class of biomaterials that hold a high water content potentially reaching up to over 99.5% weight per volume of liquid. The water is entrapped in pores within a three-dimensional network that forms when free polymer fibers condense, cross-link and interact with each other [1]. These polymer strands can be derived from many sources including natural and synthetic materials. Harvested from seaweed, alginate is an example of a naturally occurring polymer that has been used as a base material for hydrogel fabrication. Alginate consists of two polymers, which are D-mannuronic acid and L-guluronic acid [1,2]. When these two polymers are cross-linked, they form a 3D mesh in the presence of a cross-linking agent such as calcium chloride. Many other natural-based materials are also being used to form hydrogels, including proteins and polysaccharides such as collagen, gelatin, heparin, chitosan and agarose [3]. On the other hand, synthetic polymers are also widely used to form hydrogels. For instance, this is the case of polyethylene glycol—or PEG, in short—a synthetic polymer that is chemically stable, versatile, biocompatible and flexible [4]. Other synthetic polymers that have been used as materials for hydrogel formation include polylactic-co-glycolic acid (PLGA), polyvinyl alcohol (PVA) and polyethylene oxide (PEO) [5]. 

Another class of synthetic compounds that assemble to polymeric fibers and have been used for hydrogel formation comprises rationally designed self-assembling peptides. They consist of naturally occurring amino acids. These synthetic peptides are rationally designed, containing a characteristic sequence motif that allows for the control of the physico-chemical features of the peptide compound by change at the single amino acid level [6]. These peptides are all amphiphilic compounds, i.e., one domain, and generally the major domain, of the peptide is hydrophobic, while the remaining part, often just a single amino acid, is hydrophilic. This design allows the peptides to rapidly self-assemble into nanofibers when dissolved in an aqueous solution where the hydrophobic part is shielded within the nanofiber and the hydrophilic part forms the outer layer of the nanofiber that interacts with the ambient water molecules [6]. The ultimate control of the composition of the peptides, and hence their behavior, makes these peptides amenable to applications in drug release, tissue engineering, 3D cell culture and many others.

Whether they are synthesized or naturally derived, all mentioned materials can be used to form hydrogels without further modifications on their chemical structures. In addition, however, they can be chemically or biochemically modified to expand their native physico-chemical properties and exploit more advantageous features. One important example of the chemically modified natural material that has been used to form the hydrogel is gelatin methacryloyl (GelMA). The naturally existing gelatin undergoes a chemical modification by methacrylic anhydride to produce GelMA [7]. To form the hydrogel using GelMA, ultra-violet (UV) light is used in the presence of a photoinitiator to form a biocompatible hydrogel with tunable mechanical properties. GelMA has been prepared under highly controlled and consistent production methods, which strongly reduced batch-to-batch variations [8]. Such advances allow for further integration of GelMA in biomedical applications, as will be shown later in this paper.

A higher complexity and functionality can be achieved by forming hybrid hydrogels. Hybrid hydrogels are made by combining multiple chemically distinguished materials that can include natural and synthetic materials in addition to bioactive molecules such as proteins and peptides [9]. By selecting the materials that form the hybrid hydrogels and their relative quantities, bioactive hydrogels can be made to treat various defects. One particular example, which will be discussed in more detail in this paper, is a collagen-based hydrogel loaded with nanosilicate to treat bone defects [10]. 

The most important requirement for biomedical applications when using hydrogels made from various materials, such as the ones mentioned above, is the claim that these hydrogels are able to mimic the native extracellular matrix (ECM). ECM-like hydrogels need to have a high water content within the fibrous scaffolds and need to prove that they are suitable as 3D scaffolds and support cellular growth [11]. In addition to these properties, hydrogels are very important in the field of tissue engineering because they allow for the incorporation of bioactive molecules. Such bioactive molecules play significant roles in cell proliferation and cell signaling, which ultimately permits the fabrication of complex bioengineered tissues [12]. For example, it is crucial to consider a proper vascularization when fabricating complex bioactive constructs. Only with optimal vascularization will sufficient quantities of required oxygen and nutrients be delivered to living cells within these constructs. 

Hydrogel particles with diameters in the micrometer scale are defined as microgels. Microgels have been shown to be useful to support and improve vascular morphogenesis. Co-entrapping endothelial cells and mesenchymal stem cells in a microgel-based system demonstrated improved vasculogenic/angiogenic potential after injection of the cell-laden microgels into mice. The injected cells within the microgels assembled into vascularized microtissues with an enhanced cell survival. These kinds of cell-laden microgels could offer interesting opportunities in a clinical setting, for example when delivered into injured tissue [13]. 

Microgels can be similarly used as hydrogels, i.e., as vehicles for controlled delivery of therapeutic cells and therapeutic chemicals [14]. Therapeutic cells are defined as cells that are capable of treating a disease or a defect either directly, by expressing therapeutic proteins, or indirectly, by activating other cells, such as T lymphocytes, to harness their therapeutic activities [15,16]. In addition, depending on their intended use, the mechanical properties of hydrogels can be easily tuned to match the desirable application [17]. The ability to alter hydrogels’ mechanical properties while supplying them with the appropriate biological cues makes them the ideal medium for controlled drug release and controlled cells’ growth and behavior [18]. However, considering the size of the cells and the very small amount of the needed biological cues, such as growth and differentiation factors, bulk hydrogels tend to lack a precise control of the microenvironment surrounding incorporated cells. The diffusion of nutrients and the removal of waste is also hindered within bulk hydrogels due to their large diffusion path [19]. To overcome these challenges, microgels are offering more elegant solutions for many applications, especially in the biomedical field [20]. Since microgels are defined as hydrogel particles in the micrometer-scale, which can be precisely fabricated with predetermined sizes and shapes, they are promising tools for cell encapsulation, drug delivery, bioprinting and many other biomedical applications. Microgels, unlike bulk hydrogels, are predefined in terms of their size, shape and mechanical properties. Microgels exhibit a different behavior compared to bulk hydrogels in terms of injectability, modularity and porosity [3]. The injectability is a significant property of microgels which allows them to be administered to patients under a minimum invasive delivery. The modularity of the microgels implies that microgels of different sizes with different encapsulated bioactive molecules or living cells can be organized together to form functional biological constructs. The porosity which is observed when microgels are packed together introduces an interstitial void between the individual particles that aids cell migration in pertinent applications [3]. 

Microgels can be fabricated in numerous shapes and sizes applying many different fabrication methods. Each fabrication method has its own advantages as well as its own limitations in terms of controlling the size, shape, stiffness and monodispersity of the fabricated microgels. A fabrication method is said to be of high monodispersity if the fabricated hydrogels have a uniform size and shape with minimum variation [21]. Monodispersity is very important, in particular for drug delivery applications where the diffusivity of the drugs out of the microgels can be tuned to match desirable diffusion rates [22]. Microgels can also be used as cell-laden materials for many biomedical applications, including 3D models for cancer studies and drug discovery [23,24]. 

In this review, multiple microgel fabrication methods will be presented and discussed in sufficient detail, with the purpose of helping the reader to choose the most relevant and applicable fabrication method for the intended application. The fabrication methods are divided into two classes: top-down and bottom-up methods. Moreover, recent biomedical applications of microgels will be covered along with the materials used to form the used microgels for each application. In particular, we will highlight the use of self-assembling peptides as a biomaterial for microgel fabrication which differs substantially from their application in the form of nanoparticles [25].

## 2. Fabrication of Microgels by Top-Down and Bottom-Up Approaches

Different microgel fabrication methods have been developed and optimized over the past two decades as interest in microgels increased. These methods can be divided into four categories according to their underlying physical principles, which are micromolding methods, emulsion-based methods, shearing methods and extrusion methods [26]. Each of these fabrication approaches have their own required materials and equipment. For example, and as the name suggests, micromolding methods require the use of predesigned molds. These molds necessitate further materials and equipment in addition to prior knowledge of mold design and fabrication. Emulsion methods, on the other hand, do not require the use of molds, but entail the use of immiscible liquids, mostly oil and water, with a mixing equipment to form water-in-oil droplets. The materials and equipment needed will be clearly highlighted for each fabrication method that will be discussed below. One should make sure to have those materials and equipment available when selecting a particular fabrication method. The availability of the materials and equipment should not be the sole reason for selecting the appropriate microgel fabrication method. It is very important to know and consider the desired properties of the fabricated microgels, such as shape and size, when choosing the proper method to fabricate the microgels. Furthermore, of all the things to consider when choosing the fabrication method, the compatibility of the fabrication method with the gelation mechanism of the hydrogel and the final intended application should have the highest priority [3]. In the following, we consider the fabrication methods themselves, instead of the underlying physical principles, and we place them either as top-down or bottom-up approaches.

In the top-down approach, rationally designed masks or molds are used to produce microgels with highly controlled geometries [27,28]. Lithography is usually used in one of three forms: imprint lithography, photolithography or flow lithography. The general term “lithography” is defined as the use of a pattern that reflects the intended size and shape of the fabricated product. Lithography comprises the use of a mold, which is a material that has to be developed or cured in several steps. The mold is usually fabricated by the use of a mask juxtaposed on top of a material that can be cured or etched in order to take the shape of the mask. Masks are generally made out of silicon due to its durability and thermal stability [29]. 

To make microgels using imprint lithography, a patterned mold with negative features of the microgels should be used. The mold will hold and shape the hydrogel precursor that is poured into the well and then cured with different curing agents, such as heat, UV light or chemicals depending on the hydrogel material. After that, the cured microgels are extracted out of the mold and collected. This method was first implemented to fabricate microgels by the developers of the Particle Replication in Non-wetting Templates (PRINT) method [28]. In this method, the molds that holds the hydrogel precursor and give the shape to the microgels are made of photocurable perfluoropolyether (PFPE). Using these molds, microgels can be made with highly controlled size and shape with resolution in the nanometer-scale. Moreover, microgels are fabricated using PRINT without the need for the harsh processing steps that are usually required when traditional molds are used [28]. Using PRINT and DNA, therapeutic proteins and other bioactive materials can be safely encapsulated, making this method attractive for various biomedical applications [28]. This method also allows for the fabrication of microgels with non-spherical shapes. Shapes such as rods and disks are increasingly being considered for some applications, especially for drug release, as their diffusion, cell intake and surface adhesion properties are different from those of the spherical particles [30]. 

Photolithography is another lithography method that can be used to fabricate microgels, and it is defined as using light, which passes through a photomask to pattern a photosensitive polymer [31]. Instead of using a physical mold to give the microgels their size and shape, light is used as the template in photolithography. For materials where gelation can be triggered using UV light, microgels can be fabricated using photolithography with the light source being UV light [27]. Photomasks made of treated polydimethylsiloxane (PDMS) were used to fabricate cell-laden microgels [32]. 

Photolithography can be used in continuous production modes called flow lithography and stop-flow lithography [27]. Photomasks are also used in flow lithography, where they are usually combined with magnifying lenses to adjust the size of the fabricated microgels and minimize the material used in the photomask. Therefore, when comparing the other two lithography methods, a higher throughput is possible when using flow lithography [33]. This method was used to fabricate poly (ethylene glycol) (PEG) microgels [34]. The microgels were fabricated in a highly reproducible manner with a width, thickness and length of 90 µm, 30 µm and 180 to 270 µm, respectively. These microgels enabled single-fluorescence detection of DNA oligomers when combined with the appropriate probes [34]. Stop-flow lithography requires the use of a microfluidic device that is highly sensitive to changes in flowrates and pressure, so that the fluids inside the microfluidic device can shift from the flowing to the stationary state instantaneously [35]. Stop-flow lithography has been used to fabricate complex-shaped microgels. In one example, soft and temperature-responsive microgels were fabricated in a snowflake shape with a height of about 88 µm [35]. Many recent improvements in flow lithography aim to increase the throughput and the complexity of the fabricated microgels, and stop-flow lithography is one of these improvements. In one study, a multichannel microfluidic device was combined with a powerful LED UV source to achieve a two order of magnitude increase in the fabrication rate of this stop-flow lithography when compared to traditional flow lithography [36]. Four-dimensional (4D) microgels can also be fabricated using stop-flow lithography. In one study, 4D PEG microgels of non-spherical shapes were fabricated with incorporated time dimension using flow lithography [37]. By using precursor fluids of different densities and then allowing the denser fluids to settle by gravity, the 4D microgels were created by exposing the precursor fluids to patterned UV light with a throughput of about 3600 particles per hour [37]. 

In general, lithography methods enable the fabrication of microgels with highly controlled sizes and shapes. The microgels can then be used for cell encapsulation without the need to use other materials such as surfactants and oils, which are required for the emulsion-based fabrication methods [38,39]. On the other hand, lithography methods have several disadvantages, including the need to physically remove the fabricated microgels out of the molds once the fabrication is completed. The physical removal of the molds can lead to deformation of the fabricated microgels if not done properly. Depending on the material used to form the microgels, the surface of the molds must be properly treated to allow for the release of the formed microgels [32]. For example, agarose microgels which are sugar-based require a different mold surface treatment than protein-based microgels such as those made of gelatin [32]. Therefore, this surface treatment requirement prevents the usefulness of the method. Imprint lithography and photolithography are both fabrication methods that only allow for the batch production of microgels, making them less desirable when a high throughput is required. In addition, fabricating the molds and masks can be time-consuming and usually requires curing agents and the use of cleanrooms, as for mask fabrication. Another disadvantage of lithographic fabrication methods is the need to use UV light. Because of that, only UV-curable materials can be used to fabricate microgels using photolithographic methods. Moreover, the treatment with UV light can be detrimental for the fabrication of cell-laden microgels, as it has been reported that cells can be damaged by UV light [40,41]. To overcome this problem, some groups worked on improving the crosslinking techniques by using visible light [40,41,42]. So far, the flow-lithography technique with the use of visible light curing seems the most promising approach among the top-down ones.

Mechanical fragmentation is another example of a top-down method. In this method, a bulk hydrogel that is already preformed gets broken down into microgels through applied mechanical forces. One way to do this is by simply using a blender to break down a solid block of gelatin hydrogel into microgels [43]. The size of the microgels that are fabricated using a customer-grade blender operating at “pulse speed” depends on the duration of the blending. Microgels with average diameter of 55 µm were obtained when the blending duration was 120 s. The size of the microgels varies inversely with the time of blending [43]. 

Another way to mechanically produce microgels from a bulk hydrogel is by forcing the bulk hydrogel through a mesh with pores in the micrometer-scale. In a cylinder apparatus, a piston was used to extrude the bulk hydrogel through micronic mesh, resulting in the fabrication of microgels with diameters of 15 to 30 µm [44]. The major advantage of using this method is the simplicity and the speed by which the microgels are obtained. This method is mostly applicable where the monodispersity of the fabricated microgels is not of importance. Microgels fabricated using this method were used as injectable cell culture scaffolds [44]. 

Conversely, in the bottom-up approach, microgels are fabricated with a high control of the individual building blocks in a scalable manner, in order to then be assembled into complex constructs for various biomedical applications [45]. These methods include emulsion-based methods, electrohydrodynamic spraying and microfluidic-based methods. One of the simplest methods to fabricate microgels that does not require specialized equipment is the batch-emulsion method [3]. In this method, a hydrogel precursor is combined using a mechanical mixing method with an immiscible oil in the presence of a stabilizing surfactant in the same beaker. Droplets of the hydrogel precursor can be formed by homogenizing the two fluids, resulting in a water-in-oil emulsion. For example, gelatin microgels were generated in a beaker by adding gelatin solution to soy oil and stirring the solution for two minutes. The oil was then removed by centrifuging the emulsion for three minutes, followed by washing the microgels with phosphate buffered saline (PBS) [46]. The size of the fabricated microgels can be controlled by adjusting the time and the mixing speed [3]. This method is probably the most used method across the literature because it is fast, cost-effective, straightforward and does not require sophisticated equipment to be carried out. Batch emulsion can be used to encapsulate bioactive molecules as well as cells [46,47,48]. Although the batch emulsion method is fast and simple, it has as major trade-off: the non-monodispersity of the fabricated microgels. Another unavoidable limitation of this method is the intensive washing steps required to remove the oil used during the fabrication.

Electrohydrodynamic spraying is an extrusion-based approach that is less popular than the other bottom-up methods. In this method, a voltage is applied at the tip of a syringe that is loaded with a hydrogel precursor, and droplets of the hydrogel precursor form when the surface tension is overcome by the applied voltage [49]. Alginate microgels have been fabricated using this method where the generated droplets fall into a solution of calcium chloride to induce gelation [50,51]. Furthermore, polyethylene glycol (PEG) has been used to fabricate microgels using this method, with particles with diameters between 70 and 300 µm [52]. One of the advantages of this method is that it can be used for cell encapsulation [53]. However, low monodispersity is reported [52]. 

**Figure 1:**
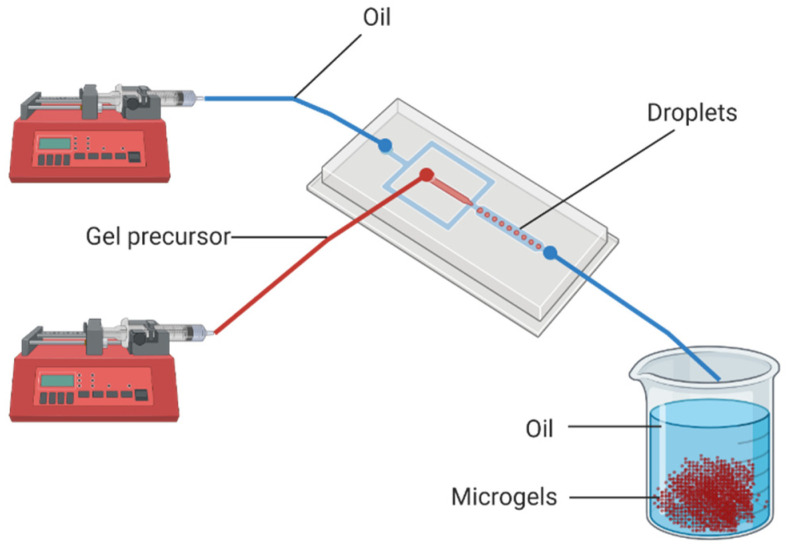
Microfluidic emulsion set up. The two immiscible fluids (water and oil) are pumped out of the syringes into the microfluidic chip. The relative flowrates of the two fluids, as well as the geometry of the microfluidic chip, dictate the size of the droplets generated in the chip. The droplets transform into microgels, which are then collected and washed to remove excess oil.

Microgels can also be made using microfluidic emulsion, which is also referred to as droplet microfluidics. In this method, a flow-focusing device, usually a chip, is used to combine two immiscible fluids, with one being the hydrogel precursor and the other being oil, to form droplets of the hydrogel precursor in oil, as illustrated in Figure 1 [54]. The size and the exit frequency of these droplets are controlled mainly by the geometry of the intersection where the droplets form and the relative flowrates of the two fluids. When the flowrates are set to appropriate levels, droplets can be produced with a diameter as small as 5 µm and as big as 500 µm, with a very low variation in the particle size of the same batch [55,56,57]. The ability to control the size and shape of the droplets, and hence of the microgels, makes microfluidics the method of choice for many applications. On the other hand, microfluidic emulsion has to overcome a few intrinsic limitations and challenges.

**Figure 2:**
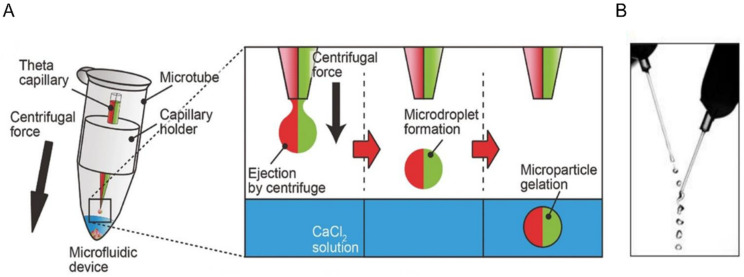
(**A**) The centrifugal microfluidic device consisting of a microtube, a Theta capillary holding the alginate solution, a capillary holder and calcium chloride solution. When centrifugal force is applied, droplets of the alginate solution fall into the CaCl_2_ solution, where gelation takes place. (**B**) The in-air microfluidic working principle is shown here as two jets of two reactive liquids that meet to form droplets which are then transformed to microgels. Upon the contact of the two jets, gelation begins, and the droplet transforms into a microgel particle. Panel (**A**) has been reproduced with a permission from Wiley [59] and Science Advances for Panel (**B**) [58].

One of the disadvantages of using traditional microfluidics to synthesize microgels is the need for deep cleaning of the microfluidic device prior the beginning of the microgel fabrication. In addition, intensive washing is usually performed to remove the oil excess from the collected microgels. To overcome these obstacles, several oil-less microfluidics methods were developed [58,59]. One of these solutions is to use a centrifugal microfluidic device to make alginate microgels, as depicted in Figure 2A [59]. In this method, the hydrogel precursor is forced out of a capillary by centrifugal force. Droplets form and drop into the CaCl_2_ solution, under which they assemble into a hydrogel. The microgels can then be collected from the bottom of the tube. The size of the droplets generated depends on the magnitude of the applied centrifugal force and the size of the capillary head. Although this method eliminates the need for oil and the associated washing steps to remove the excess oil, it requires a manual reloading of the hydrogel precursor, in addition to being a low throughput fabrication method. 

Another method in which the need for oil is eliminated is the in-air microfluidics. In this method, the hydrogel precursor is jetted from a nozzle to form droplets, which meet and combine with droplets from a second reactive liquid: due to the surface tension between the two liquids they then combine into spherical microgels, as seen in Figure 2B [58]. This method eliminates the need for a microfluidic chip and consequently increases the production rate of microgels up to 100 times when compared to the traditional chip-based microfluidics. However, the positions of the nozzles have to be adjusted and maintained throughout the fabrication period to achieve an impact angle of 25°.

## 3. Microgels in the Delivery of Therapeutic Agents

**Figure 3:**
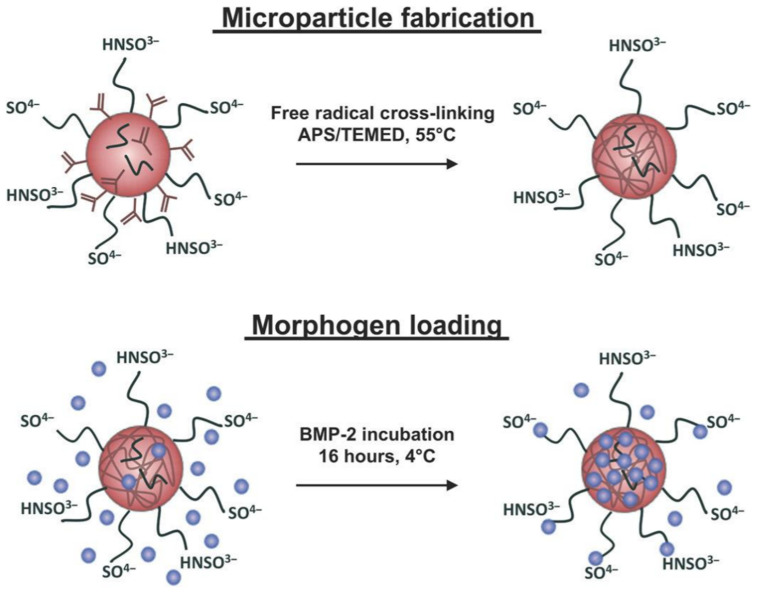
Fabrication of heparin microgels and their loading with human bone morphogenetic protein-2. Heparin methacrylamide was used to fabricate the microgels in a water-in-oil emulsion. APS/TEMED refers to the free radical initiators ammonium persulfate (APS) and tetramethylethane-1,2-diamine (TEMED), respectively. HNSO^3−^ and SO^4−^ are the sulfate groups on heparin that bind to BMP-2. The figure has been reproduced with permission from Science Advances [62].

Microgels have been used as delivering vehicles to carry bioactive therapeutics. Because they are controlled in size, shape and stiffness, the diffusivity of encapsulated drugs can be precisely regulated as well. Single or multiple hydrogel-based materials can be used to achieve a desirable degradability when interactions between the drug and the hydrogel-based materials are present. A sustained drug release profile can be achieved when microgels are delivered via injection, in a minimally invasive manner, to the intended site where biomolecules, such as growth factors, are needed [60]. One must consider all possible interactions, such as hydrophobic and hydrophilic interactions, between the drug to be delivered and the hydrogel-based material of choice. Exploiting the natural affinity of some bioactive molecules and hydrogel-based materials can lead to more effective medical treatment methods. One example of such interaction is the affinity between heparin and the Bone Morphogenetic Proteins (BMPs) [61]. Heparin methacrylamide microgels were fabricated using a batch emulsion, and then loaded with human BMP-2 to treat large bone defects (Figure 3) [62]. The heparin microgels were able to maintain the bioactivity of BMP-2, enhance BMP-2 retention and improve bone formation in large bone defects, clearly showing advantages over the standard clinical method, which relies on collagen sponge scaffolds for the localization of the BMP-2 delivery [62]. 

Regarding the fabrication method, the heparin methacrylamide solution was prepared by dissolving heparin methacrylamide in PBS. Promptly after gelation, the solution was added in a drop-by-drop fashion to a solution of corn oil and polysorbate 20 where the latter is a surfactant. Homogenization was then carried out on ice and the homogenized solution was obtained using a homogenizer for 5 min. Excess oil was then removed via centrifugation after several washing steps with acetone rather than water [63]. Since the microgels were fabricated using the batch emulsion method, they consequently had low monodispersity and therefore resulted in variations of the BMP-2 release profile. Microgels with high monodispersity will undoubtedly result in a more uniform and predictable drug release profile. 

BMP-2 was also combined with osteogenic bone marrow mesenchymal stem cells (hMSCs) and encapsulated in microgels made of polyvinyl alcohol (PVA) and fabricated using a chip-based microfluidic device [64]. The size of the fabricated microgels was controlled by adjusting the flowrates of the incoming solutions to obtain microgels of sizes between 100 µm and 200 µm. The encapsulated cells maintained their viability, while the BMP-2 improved the osteogenic differentiation of the encapsulated hMSCs.

Furthermore, microgels are suited to deliver drugs to the pulmonary system, unlike bulk hydrogels, which could block the airways. This risk is due to the fact that bulk hydrogels are larger in size and can physically clog the airways. In one example, microgels were produced by using polyethylene glycol (PEG) that was peptide-functionalized. The microgels were fabricated using a batch emulsion where the PEG solution was homogenized with silicone oil by vortexing for 10 min at the highest speed setting. The microgels were then cross-linked by placing the emulsion under UV light for 30 min [65]. The desirable size of the fabricated microgels, that are intended for pulmonary drug delivery, is 5 µm for bronchial delivery and as low as 0.5 µm if deeper penetration is needed [66,67]. Microgels were also used to deliver other important bioactive materials such as insulin and the vascular endothelial growth factor (VEGF). They showed effectivity and reliability in the treatment of diabetes and myocardial infarctions, respectively [68,69,70,71].

**Figure 4:**
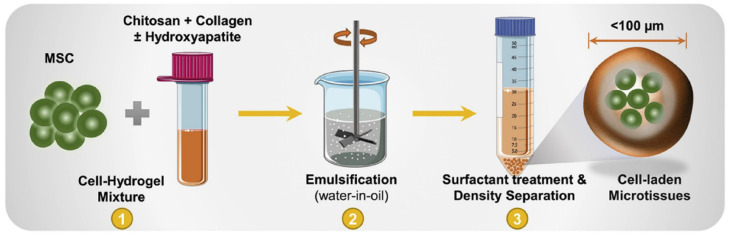
Fabrication of mesenchymal stromal cells (MSC) cell-laden chitosan-collagen microgels. Emulsification was used to generate the microgels with a particle diameter of about 100 µm. The microgels were able to maintain cell viability for up to 21 days. The figure has been reproduced with permission from Elsevier [74].

In addition to being used as delivering vehicles for bioactive materials, microgels have also been used to deliver therapeutic cells in various medical treatments due to their ability to physically isolate the delivered cells from immune cells [72,73]. For example, chitosan-collagen microgels were used to encapsulate and deliver mesenchymal stromal cells (MSC) to promote bone regeneration in defected bones [74]. The microgels were produced using a water-in-oil emulsion, as seen in Figure 4.

Microgels have been used to deliver liposomes containing bioactive molecules. One example of importance is the encapsulation of kartogenin, which is a small molecule that has chondrogenic effect on mesenchymal stem cells [75]. An effective delivery method of kartogenin was developed by integrating kartogenin-containing liposomes into microgels made of gelatin methacryloyl (GelMA) [76]. Liposome vesicles made of phospholipid bilayers [77] were used here to overcome the low solubility of kartogenin in water. The GelMA microgels were fabricated using microfluidic methods to generate GelMA-in-oil droplets, which were then crosslinked using UV light [76]. The effectiveness of this method was tested in vitro and showed a significant promotion of mesenchymal stem cells to differentiate to chondrocytes [76]. In addition, the microgels showed a reduction of osteophyte and prevented the degeneration of the articular cartilage [76].

**Figure 5:**
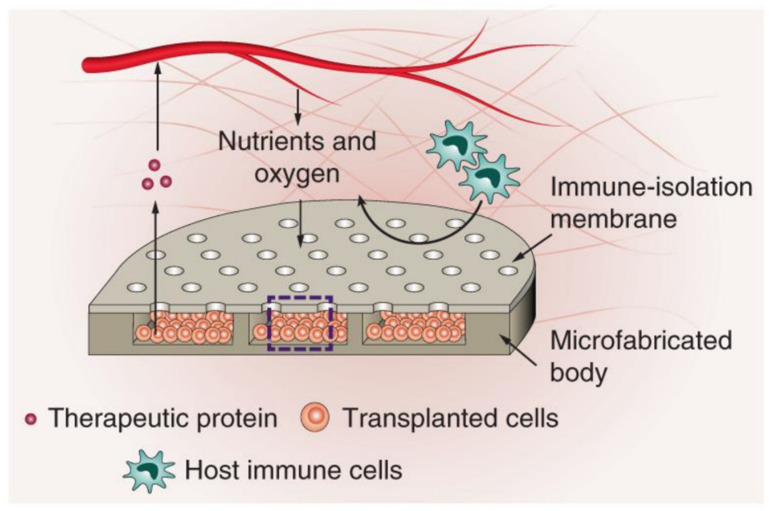
Design and working principle of a biocompatible device housing therapeutic cells. The cells are embedded in alginate microgels allowing for the intake of nutrients and oxygen, removal of waste and secretion of therapeutic proteins while keeping the host’s immune cells from entering the device. The figure has been reproduced with permission from Springer Nature [78].

A very recent development in cell therapy allowed for the long-term functioning of therapeutic cells encapsulated in alginate microgels without the need for immunosuppression [78]. This was achieved by trapping the encapsulated cells inside the microgels in an engineered porous device, as depicted in Figure 5. By controlling the size of the pores, immune cells were kept away from the encapsulated cells, allowing them to continue secreting their therapeutic proteins. This method was proven effective when a normal level of blood sugar was achieved and maintained for more than 75 days after pancreatic islets were encapsulated and introduced in diabetic mice [78].

Because this method relies on the diffusion of oxygen and nutrients from nearby blood vessels, only a few layers of cell-laden microgels can be packed before the diffusion limit is reached. One innovative solution to this problem is to use oxygen-generating microgels which have emerged as a new method that can be used to engineer more complex scaffolds while maintaining a sufficient oxygen level for the encapsulated cells. In one study, polycaprolactone and pluronic F-127, which are clinical-grade polymers, were used to form oxygen-generating microgels [79]. Calcium peroxide was incorporated with the two polymers as the oxygen source. The microgels were fabricated using electrohydrodynamic spraying, 12 kV was used as the voltage, and the generated droplets were collected in a methanol solution placed in a plate. The microgels were then collected and washed to remove the methanol. The microgels increased the cell survival of chondrocytes when incorporated in a GelMA scaffold. Therefore, this technology can be used to ensure the continuous supplement of a sufficient level of oxygen to cells allowing for the fabrication of biofunctional complex constructs. 

## 4. Microgels in Biofabrication

Biofabrication is defined as the rational and organized combination of biomaterials, bioactive molecules and living cells to produce biologically functioning products [80]. Biofabrication can have two forms, which are bioprinting and bioassembly [81]. First, bioprinting is defined as the spatial organization of bioinks consisting of live cells, biomaterials and bioactive molecules to build 3D biofunctional constructs [82]. On the other hand, bioassembly is defined as the automated organization of individual building blocks resulting in a biofunctional constructs when rationally assembled [80]. Microgels can be bioprinted alone or in combination with bulk hydrogels to build complex 3D biofunctional structures. The combination of microgels with bulk hydrogels is a modular approach that allows for the incorporation of various biomaterials to enable the fabrication of multifunctional tissues. In one example of bioprinting microgels, microgels with a diameter less than 40 µm containing encapsulated single cells were first fabricated by microfluidics technology using PEG [83]. The microgels were then bioprinted in combination with injectable bulk hydrogel to make 3D biostructures [83]. The concentration of the microgels within the bulk hydrogel was considered in order to match the cell concentration of different tissues in the body. 

**Figure 6:**
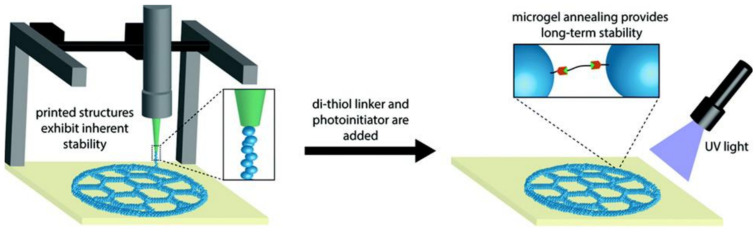
Setup of 3D bioprinting of PEG microgels. The extrusion of the microgels requires low applied force, allowing for cell incorporation while maintaining cell viability. Crosslinking the microgels via thiol-ene reaction allows for long-term stability. The figure has been reproduced with permission from the Royal Society of Chemistry [84].

In addition, when combined with bulk hydrogels, microgels can also be compacted and printed alone to create 3D structures, as seen in Figure 6 [84]. In this case, the shown printed structures maintained their shape by the use of di-thiol linkers and photoinitiators. Furthermore, bioinks made of compressed or jammed microgels showed shear-thinning behavior where they flow as a liquid when external force is applied, and behaved as solid when the force is lifted [85]. It is important to consider the osmotic pressure of the microgels that encapsulate living cells, as low osmotic pressure will preserve the morphology of the encapsulated cells and maintain their viability while allowing nutrients to diffuse to the encapsulated cells [86]. Low osmotic pressure can be obtained by using a low concentration of the hydrogel-based material when fabricating the microgels. Therefore, cell-encapsulating microgels can be bioprinted into stable constructs using layer-by-layer or gel-in-gel bioprinting methods while maintaining high cell viability [85]. 

Very recently, microgels of various shapes, including squares and triangles, were assembled into constructs by using characteristically shaped hollow microcages [87]. In addition of being hollow, the microcages are rigid and stackable, which permits the scalability and manual assembly of complex structures very easily. The microcages were loaded with GelMA microgels, which were then loaded with biological cues, including cues from vascular endothelial growth factor (VEGF) and platelet-derived growth factor (PDGF). Human umbilical vein endothelial cells (HUVECs) and human mesenchymal stem cells (HMSCs) showed instructed migration following the gradient of the cues when co-cultured and seeded on the top layer of the microcage. Moreover, the cells were able to proliferate at a higher rate in scaffolds loaded with microgels compared to scaffolds with bulk hydrogels. 

One of the main challenges of assembling large biofunctional constructs, as mentioned in the introduction of this paper, is the vascularization challenge, as the diffusion in avascular tissues is limited to about 200 µm [88]. Many methods have been developed to induce the formation of blood vessels to treat vascular disorders or maintain cell viability within tissue-engineered scaffolds [89]. Microgels are increasingly considered as a viable solution to tackle the vascularization challenge when used to encapsulate endothelial cells which are known to have neovascularization activity [90]. In one study, type I collagen was combined with ultra-long DNA and used to encapsulate human umbilical vein endothelial cells (HUVECs) with the addition of VEGF [91]. Ultra-long single-stranded DNA that is negatively charged was used to enable a faster gelation of the collagen, which is positively charged. The microgels with the encapsulated cells and the VEGF were fabricated using microfluidic emulsion, which preserved the collagen biochemical and biodegradable properties. When tested in vivo, the microgels showed strong angiogenic activity, which significantly improves liver regeneration and skin repair [91]. 

Another unique usage of microgels in bioprinting is to print with cell-only bioinks within a supporting bath made of microgels [92]. The shear-thinning property of jammed microgels, as mentioned before, allows for the bioprinting of cells by inserting a moving needle that injects the cells as it moves within the volume of microgels, creating a stable 3D structure of the printed cells. hMSCs and other cell types were printed within a bath of alginate microgels with higher resolution, observed when smaller microgels were used [92]. 

## 5. Microgels from Ultrashort Self-Assembling Peptides 

One major advancement in the field of microgels is the ability to rationally design and synthesize the building blocks of the material that is used to generate the microgels. Traditional materials, which include naturally-derived materials such as chitosan [93] gelatin [94] and sodium alginate [95], are prone to possible contamination of chemical or biological agents that could lead to immunogenicity [96]. In addition, the harvest and purification of those materials can include the use of toxic reagents which could possibly harm the cells, making these materials unattractive. To eliminate these obstacles, self-assembling peptides that are rationally designed and synthetic, made of natural amino acids, can be used as a better alternative with additional benefits, such as precisely controlled biodegradability rates and tunable antimicrobial activity [97].

Ultrashort self-assembling peptides are a class of rationally designed peptide compounds with sizes ranging from 3 to 7 amino acids [6,98]. The peptides are amphiphilic, which means that they contain domains of both hydrophobic and hydrophilic amino acids. The amino acids are arranged in such way that the peptide sequence contains a majority of aliphatic nonpolar amino acids as the hydrophobic part, whereas the hydrophilic domain comprises polar amino acids, often at the C-terminus and just containing the single charged amino acid lysine [99]. This arrangement gives the peptide a hydrophobic tail that decreases in hydrophobicity as it gets closer to the C terminus, where it connects to a hydrophilic head. Because of this design, the peptides self-assemble into nanofibers when dissolved in water to form hydrogels. The properties of the formed hydrogels, such as gelation time and strength, can be controlled by the choice of the amino acids of the peptide, as well as the length of the peptide [6,98]. The amino acids used to make these peptides are natural amino acids such as leucine, valine, alanine and glycine [6]. 

The self-assembling peptides form nanofibers that are hydrophobic in the core and hydrophilic in the outer shell [6,100]. Due to the hydrophilicity, the nanofibers attract water and form a network resulting in a hydrogel that has a water content as high as 99.9% (w/v) [101]. The hydrogels made from the peptides are biocompatible, thermally stable and mechanically stiff [98]. In addition, the well-defined chemistry of the hydrogels makes them suited to be used as scaffolds that allow pluripotent stem cells to maintain pluripotency and then be used in bioprinting or as organoid cultures [102,103,104]. Cell viability was also shown with primary human corneal cells, as well as epithelial cells, when seeded onto the peptide hydrogel [105]. Together with the excellent properties reported for bulk peptide hydrogels from ultrasort self-assembling peptides, peptide microgels have also demonstrated additional excellent properties.

**Figure 7:**
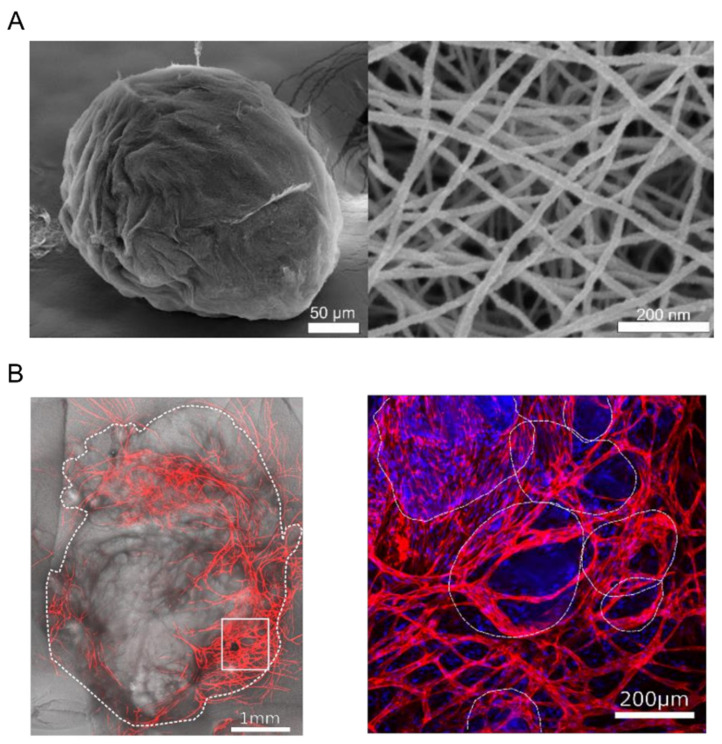
(**A**) SEM image of a single microgel (left) showing the round spherical shape and the network of fibers (right) resulting from the self-assembling of the ultrashort peptides. The figures have been adopted from [108]. (**B**) Vascularization of 3D bulk hydrogel with embedded endothelial cell-laden microgels. Anti-CD31 Alexa Fluor (red) was used for the detection of endothelial cells. 4′,6-diamidino-2-phenylindole (DAPI) (blue) was used for the detection of nuclei. The right image is a zoom-in of the left image, and both images show that the vascular network originated from the embedded microgels and spread to the rest of the bulk hydrogel.

Microgels have been generated from solutions containing self-assembling peptides, using water-in-oil emulsion in the presence of salt to enhance gelation and showing great control over the size of the microgels, as well as maintaining cell viability when using them for cell encapsulation [106,107]. Recently, the already described class of self-assembling ultrashort peptides were used to synthesize microgels. The microgels were produced using the traditional oil-based microfluidic chip. The fabricated microgels ranged in diameter between 300 and 350 µm (Figure 7A), and were used as cell-laden materials carrying human umbilical endothelial cells (HUVECs), which were combined in a bulk hydrogel with human dermal neonatal fibroblast [106]. Combining microgels and their cell content within the bulk hydrogel resulted in the formation of a vascular network only 20 days after culturing, as shown in Figure 7B [108].

Besides microgels, submicron peptide hydrogel particles, which are referred to as nanoparticles made of self-assembling peptides, were fabricated with a diameter of about 73 nm, using a flow-focusing microfluidic platform together with solutions of self-assembling ultrashort peptides [109]. The nanoparticles were produced by self-assembling peptides that aggregated in water as a result of the pressure exerted on the peptide solution in the presence of two streams of 50% (*v*/*v*) ethanol solution. In addition, the optimal flowrate ratio was found to be 1:10, as for peptide to ethanol. These nanoparticles made of hydrogels are biocompatible and biodegradable, which will allow them to be used in delivering therapeutic agents without any toxicity for the body. The nanoparticles made of hydrogels are also mechanically stable and can withstand bioprinting conditions [109]. 

Self-assembling peptides have also been used in 3D bioprinting [110]. Stable 3D scaffolds were fabricated using ultrashort self-assembling peptides in a vacuum system that utilizes a robotic arm [108]. A possible biomedical application for this method is the promotion of muscle repair in injured skeletal muscles, which was performed with muscle myoblast cells [111]. The bioprinted 3D structures have proven biocompatible and maintained cell viability post bioprinting. Both microgels and nanoparticles made from ultrashort self-assembling peptides can be used in bioprinting applications, as mentioned before [109,110]. The flexibility of combining different forms of gels made from ultrashort self-assembling peptides, i.e., bulk peptide hydrogels and peptide microgels as well as peptide nanoparticles, renders this class of self-assembling peptides a very attractive platform system for a variety of biomedical applications and for applications in regenerative and personalized medicine.

**Figure 8:**
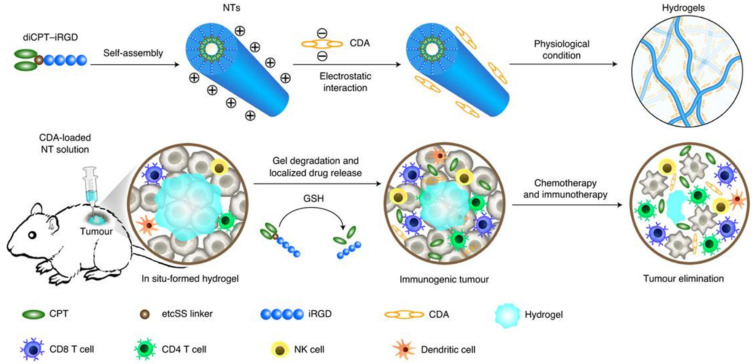
Principle and working mechanism of the self-assembling therapeutic peptide diCPT-iRGD. The resulting hydrogel releases camptothecin (CPT) when reacting with glutathione (GSH) and releases c-di-AMP (CDA) as the hydrogel degrades. This stimulates the STING pathway and leads to the infiltration of activated immune cells, thus leading to the elimination of cancerous cells. The figure has been reproduced with permission from Springer Nature [113].

Hydrogels made of self-assembling peptides are also used in cancer treatment [112]. In a very recent study, a self-assembling peptide consisting of the hydrophilic peptideinternalizing RGD peptide (iRGD) and the hydrophobic anticancer drug camptothecin (CPT) was used to form a chemoimmunotherapeutic hydrogel (Figure 8) [113]. The result is an amphipathic molecule that self-assembles into nanotubes which immediately form a hydrogel when subjected to physiological conditions. Due to the charged surface of the nanotubes, it is possible to load the nanotubes with therapeutic chemicals using the electrostatic interactions. In this case, the negatively charged c-di-AMP (CDA), which is a stimulator of interferon genes (STING) agonist, accumulates on the surface of the nanotubes. When the hydrogel is applied to tumors, the gel degrades and releases the anticancer therapeutics and the STING pathway is activated. This method was applied and tested in vivo and showed effective regression in established tumors [113]. Although no microgels were fabricated in this particular study using the diCPT-iRGD peptide, we believe that a higher effectiveness could have been achieved if microgels were used instead of the injection of the bulk hydrogel. 

## 6. Conclusions and Future Perspective

In this review, we discussed in detail the fabrication methods of microgels including both top-down and bottom-up approaches. Each of the fabrication methods has its own biomedical applications as well as a set of requirements, which were both discussed. We have also discussed a variety of materials used to form the microgels, including naturally-derived, synthetic polymeric and synthetic but natural materials and the mixture of more than one material to form hybrid hydrogels. We believe this field of biotechnology will continue to grow with the emergence of new fabrication methods as well as new hydrogel-based materials. We expect more bottom-up approaches to be developed, as they offer more control on the building blocks of the fabricated microgels.

Applications that involve microgels are expected to expand as the fabrication methods become more cost-effective. We also foresee an increase in the quality of the fabricated microgels in terms of monodispersity, which in turn will attract more applications, especially in the field of controlled drug release. The involvement of microgels in diverse biomedical applications should also increase as bioprinting technologies advance. With highly precise fully automated robotic 3D bioprinters, more complex tissue-engineered constructs are expected to increase in availability and complexity to hopefully reach the level of being able to fabricate fully 3D bioprinted functional organs. Such complexity will be obtained when microgels of different diameters are combined to form a complex biofunctional scaffolds. Cell-laden microgels can be combined with nanoparticles containing signaling molecules—for example growth factors, besides others. By adjusting the degradability of the fabricated microgels, multiple drugs can be released with different releasing profiles to treat diseases and defects better than what current clinical methods can do. Microgels will be increasingly used in the transition to personalized medicine. In particular, with advances in protein chemistry and synthetic biology, microgels offer the optimal method in the drug discovery field. Furthermore, cell-laden microgels encapsulating different types of cells can be arranged in layers to resemble the cellular layer order seen in native tissues. 

Lastly, the commercial-scale production of microgels for biomedical applications with high control of size, shape and mechanical properties remains a challenge. For pharmaceutical applications, such as drug delivery, commercial-scale production must increase the production or the throughput rate while maintaining the accuracy of the lab-scale version of the fabrication method. We expect more synthetic materials to be used to form microgels, to be developed with chemical properties that mimic the native polymers found in the ECM while eliminating the source and batch variations which are usually associated with naturally derived materials.

## Data Availability

No new data were created or analyzed in this study. Data sharing is not applicable to this article.

## References

[1] Dai Z., Ngai T. (2013). Microgel particles: The structure-property relationships and their biomedical applications. J. Polym. Sci. Part A Polym. Chem..

[2] Lee K.Y., Mooney D.J. (2012). Alginate: Properties and biomedical applications. Prog. Polym. Sci..

[3] Daly A.C., Riley L., Segura T., Burdick J.A. (2019). Hydrogel microparticles for biomedical applications. Nat. Rev. Mater..

[4] Bohn P., Meier M.A.R. (2020). Uniform poly(ethylene glycol): A comparative study. Polym. J..

[5] Munim S.A., Raza Z.A. (2019). Poly(lactic acid) based hydrogels: Formation, characteristics and biomedical applications. J. Porous Mat..

[6] Hauser C.A.E., Deng R., Mishra A., Loo Y., Khoe U., Zhuang F., Cheong D.W., Accardo A., Sullivan M.B., Riekel C. (2011). Natural tri- to hexapeptides self-assemble in water to amyloid β-type fiber aggregates by unexpected α-helical intermediate structures. Proc. Natl. Acad. Sci. USA.

[7] Yue K., Trujillo-de Santiago G., Alvarez M.M., Tamayol A., Annabi N., Khademhosseini A. (2015). Synthesis, properties, and biomedical applications of gelatin methacryloyl (GelMA) hydrogels. Biomaterials.

[8] Zhu M., Wang Y., Ferracci G., Zheng J., Cho N.-J., Lee B.H. (2019). Gelatin methacryloyl and its hydrogels with an exceptional degree of controllability and batch-to-batch consistency. Sci. Rep..

[9] Palmese L.L., Thapa R.K., Sullivan M.O., Kiick K.L. (2019). Hybrid hydrogels for biomedical applications. Curr. Opin. Chem. Eng..

[10] Xavier J.R., Thakur T., Desai P., Jaiswal M.K., Sears N., Cosgriff-Hernandez E., Kaunas R., Gaharwar A.K. (2015). Bioactive Nanoengineered Hydrogels for Bone Tissue Engineering: A Growth-Factor-Free Approach. ACS Nano.

[11] Caliari S.R., Burdick J.A. (2016). A practical guide to hydrogels for cell culture. Nat. Methods.

[12] Drury J.L., Mooney D.J. (2003). Hydrogels for tissue engineering: Scaffold design variables and applications. Biomaterials.

[13] Torres A.L., Bidarra S.J., Vasconcelos D.P., Barbosa J.N., Silva E.A., Nascimento D.S., Barrias C.C. (2020). Microvascular engineering: Dynamic changes in microgel-entrapped vascular cells correlates with higher vasculogenic/angiogenic potential. Biomaterials.

[14] Li J., Mooney D.J. (2016). Designing hydrogels for controlled drug delivery. Nat. Rev. Mater..

[15] Roh K.-H., Nerem R.M., Roy K. (2016). Biomanufacturing of Therapeutic Cells: State of the Art, Current Challenges, and Future Perspectives. Annu. Rev. Chem. Biomol. Eng..

[16] Sadelain M., Rivière I., Riddell S. (2017). Therapeutic T cell engineering. Nature.

[17] Arakaki K., Kitamura N., Fujiki H., Kurokawa T., Iwamoto M., Ueno M., Kanaya F., Osada Y., Gong J.P., Yasuda K. (2010). Artificial cartilage made from a novel double-network hydrogel: In vivo effects on the normal cartilage and ex vivo evaluation of the friction property. J. Biomed. Mater. Res. A.

[18] He W., Reaume M., Hennenfent M., Lee B.P., Rajachar R. (2020). Biomimetic hydrogels with spatial- and temporal-controlled chemical cues for tissue engineering. Biomater. Sci..

[19] Axpe E., Chan D., Offeddu G.S., Chang Y., Merida D., Hernandez H.L., Appel E.A. (2019). A Multiscale Model for Solute Diffusion in Hydrogels. Macromolecules.

[20] Wang Y., Guo L., Dong S., Cui J., Hao J. (2019). Microgels in biomaterials and nanomedicines. Adv. Colloid Interface Sci..

[21] Kim D.-Y., Jin S.H., Jeong S.-G., Lee B., Kang K.-K., Lee C.-S. (2018). Microfluidic preparation of monodisperse polymeric microspheres coated with silica nanoparticles. Sci. Rep..

[22] Xu Q., Hashimoto M., Dang T.T., Hoare T., Kohane D.S., Whitesides G.M., Langer R., Anderson D.G. (2009). Preparation of monodisperse biodegradable polymer microparticles using a microfluidic flow-focusing device for controlled drug delivery. Small.

[23] Headen D.M., García J.R., García A.J. (2018). Parallel droplet microfluidics for high throughput cell encapsulation and synthetic microgel generation. Microsyst. Nanoeng..

[24] Loessner D., Meinert C., Kaemmerer E., Martine L.C., Yue K., Levett P.A., Klein T.J., Melchels F.P.W., Khademhosseini A., Hutmacher D.W. (2016). Functionalization, preparation and use of cell-laden gelatin methacryloyl–based hydrogels as modular tissue culture platforms. Nat. Protoc..

[25] Zhang H., Zhai Y., Wang J., Zhai G. (2016). New progress and prospects: The application of nanogel in drug delivery. Mater. Sci. Eng. C.

[26] Farjami T., Madadlou A. (2017). Fabrication methods of biopolymeric microgels and microgel-based hydrogels. Food Hydrocoll..

[27] Helgeson M.E., Chapin S.C., Doyle P.S. (2011). Hydrogel microparticles from lithographic processes: Novel materials for fundamental and applied colloid science. Curr. Opin. Colloid Interface Sci..

[28] Rolland J.P., Maynor B.W., Euliss L.E., Exner A.E., Denison G.M., DeSimone J.M. (2005). Direct Fabrication and Harvesting of Monodisperse, Shape-Specific Nanobiomaterials. J. Am. Chem. Soc..

[29] Franssila S., Davis C.E., LeVasseur M.K., Cao Z., Yobas L., Tilli M., Motooka T., Airaksinen V.-M., Franssila S., Paulasto-Kröckel M., Lindroos V. (2015). Chapter 27—Microfluidics and BioMEMS in Silicon. Handbook of Silicon Based MEMS Materials and Technologies.

[30] Tuntanatewin W., Mekwatanakarn P., Zhang H., Okamura Y. (2021). Facile fabrication of elongated polymer micro/nano discs and their surface adhesiveness. J. Appl. Polym. Sci..

[31] Tiginyanu I., Ursaki V., Popa V., Makhlouf A.S.H., Tiginyanu I. (2011). 10—Nanoimprint lithography (NIL) and related techniques for electronics applications. Nanocoatings and Ultra-Thin Films.

[32] Tang M.D., Golden A.P., Tien J. (2003). Molding of Three-Dimensional Microstructures of Gels. J. Am. Chem. Soc..

[33] Dendukuri D., Pregibon D.C., Collins J., Hatton T.A., Doyle P.S. (2006). Continuous-flow lithography for high-throughput microparticle synthesis. Nat. Mater..

[34] Pregibon D.C., Toner M., Doyle P.S. (2007). Multifunctional Encoded Particles for High-Throughput Biomolecule Analysis. Science.

[35] Wolff H.J.M., Linkhorst J., Göttlich T., Savinsky J., Krüger A.J.D., de Laporte L., Wessling M. (2020). Soft temperature-responsive microgels of complex shape in stop-flow lithography. Lab Chip.

[36] Le Goff G.C., Lee J., Gupta A., Hill W.A., Doyle P.S. (2015). High-Throughput Contact Flow Lithography. Adv. Sci. (Weinh.).

[37] Paulsen K.S., Chung A.J. (2016). Non-spherical particle generation from 4D optofluidic fabrication. Lab Chip.

[38] Nichol J.W., Koshy S.T., Bae H., Hwang C.M., Yamanlar S., Khademhosseini A. (2010). Cell-laden microengineered gelatin methacrylate hydrogels. Biomaterials.

[39] Panda P., Ali S., Lo E., Chung B.G., Hatton T.A., Khademhosseini A., Doyle P.S. (2008). Stop-flow lithography to generate cell-laden microgel particles. Lab Chip.

[40] de Gruijl F.R., van Kranen H.J., Mullenders L.H.F. (2001). UV-induced DNA damage, repair, mutations and oncogenic pathways in skin cancer. J. Photochem. Photobiol. B.

[41] Noshadi I., Hong S., Sullivan K.E., Shirzaei Sani E., Portillo-Lara R., Tamayol A., Shin S.R., Gao A.E., Stoppel W.L., Black Iii L.D. (2017). In vitro and in vivo analysis of visible light crosslinkable gelatin methacryloyl (GelMA) hydrogels. Biomater. Sci..

[42] Lim K.S., Klotz B.J., Lindberg G.C.J., Melchels F.P.W., Hooper G.J., Malda J., Gawlitta D., Woodfield T.B.F. (2019). Visible Light Cross-Linking of Gelatin Hydrogels Offers an Enhanced Cell Microenvironment with Improved Light Penetration Depth. Macromol. Biosci..

[43] Hinton T.J., Jallerat Q., Palchesko R.N., Park J.H., Grodzicki M.S., Shue H.-J., Ramadan M.H., Hudson A.R., Feinberg A.W. (2015). Three-dimensional printing of complex biological structures by freeform reversible embedding of suspended hydrogels. Sci. Adv..

[44] Sinclair A., O’Kelly M.B., Bai T., Hung H.-C., Jain P., Jiang S. (2018). Self-Healing Zwitterionic Microgels as a Versatile Platform for Malleable Cell Constructs and Injectable Therapies. Adv. Mater..

[45] Du Y., Lo E., Ali S., Khademhosseini A. (2008). Directed assembly of cell-laden microgels for fabrication of 3D tissue constructs. Proc. Natl. Acad. Sci. USA.

[46] Leong W., Lau T.T., Wang D.-A. (2013). A temperature-cured dissolvable gelatin microsphere-based cell carrier for chondrocyte delivery in a hydrogel scaffolding system. Acta Biomater..

[47] Franco C.L., Price J., West J.L. (2011). Development and optimization of a dual-photoinitiator, emulsion-based technique for rapid generation of cell-laden hydrogel microspheres. Acta Biomater..

[48] Liu A.L., García A.J. (2016). Methods for Generating Hydrogel Particles for Protein Delivery. Ann. Biomed. Eng..

[49] Naqvi S.M., Vedicherla S., Gansau J., McIntyre T., Doherty M., Buckley C.T. (2016). Living Cell Factories—Electrosprayed Microcapsules and Microcarriers for Minimally Invasive Delivery. Adv. Mater..

[50] Gansau J., Kelly L., Buckley C.T. (2018). Influence of key processing parameters and seeding density effects of microencapsulated chondrocytes fabricated using electrohydrodynamic spraying. Biofabrication.

[51] Kim P.-H., Yim H.-G., Choi Y.-J., Kang B.-J., Kim J., Kwon S.-M., Kim B.-S., Hwang N.S., Cho J.-Y. (2014). Injectable multifunctional microgel encapsulating outgrowth endothelial cells and growth factors for enhanced neovascularization. J. Control Release.

[52] Qayyum A.S., Jain E., Kolar G., Kim Y., Sell S.A., Zustiak S.P. (2017). Design of electrohydrodynamic sprayed polyethylene glycol hydrogel microspheres for cell encapsulation. Biofabrication.

[53] Jayasinghe S.N., Townsend-Nicholson A. (2006). Stable electric-field driven cone-jetting of concentrated biosuspensions. Lab Chip.

[54] Anna S.L., Bontoux N., Stone H.A. (2003). Formation of dispersions using “flow focusing” in microchannels. Appl. Phys. Lett..

[55] De Geest B.G., Urbanski J.P., Thorsen T., Demeester J., De Smedt S.C. (2005). Synthesis of Monodisperse Biodegradable Microgels in Microfluidic Devices. Langmuir.

[56] Nisisako T., Torii T. (2008). Microfluidic large-scale integration on a chip for mass production of monodisperse droplets and particles. Lab Chip.

[57] Pittermannová A., Ruberová Z., Zadražil A., Bremond N., Bibette J., Štěpánek F. (2016). Microfluidic fabrication of composite hydrogel microparticles in the size range of blood cells. RSC Adv..

[58] Visser C.W., Kamperman T., Karbaat L.P., Lohse D., Karperien M. (2018). In-air microfluidics enables rapid fabrication of emulsions, suspensions, and 3D modular (bio)materials. Sci. Adv..

[59] Yoshida S., Takinoue M., Onoe H. (2017). Compartmentalized Spherical Collagen Microparticles for Anisotropic Cell Culture Microenvironments. Adv. Healthc. Mater..

[60] Lienemann P.S., Lutolf M.P., Ehrbar M. (2012). Biomimetic hydrogels for controlled biomolecule delivery to augment bone regeneration. Adv. Drug Deliv. Rev..

[61] Ruppert R., Hoffmann E., Sebald W. (1996). Human bone morphogenetic protein 2 contains a heparin-binding site which modifies its biological activity. Eur. J. Biochem..

[62] Hettiaratchi M.H., Krishnan L., Rouse T., Chou C., McDevitt T.C., Guldberg R.E. (2020). Heparin-mediated delivery of bone morphogenetic protein-2 improves spatial localization of bone regeneration. Sci. Adv..

[63] Hettiaratchi M.H., Miller T., Temenoff J.S., Guldberg R.E., McDevitt T.C. (2014). Heparin microparticle effects on presentation and bioactivity of bone morphogenetic protein-2. Biomaterials.

[64] Hou Y., Xie W., Achazi K., Cuellar-Camacho J.L., Melzig M.F., Chen W., Haag R. (2018). Injectable degradable PVA microgels prepared by microfluidic technology for controlled osteogenic differentiation of mesenchymal stem cells. Acta Biomater..

[65] Secret E., Crannell K.E., Kelly S.J., Villancio-Wolter M., Andrew J.S. (2015). Matrix metalloproteinase-sensitive hydrogel microparticles for pulmonary drug delivery of small molecule drugs or proteins. J. Mater. Chem. B.

[66] Du J., Du P., Smyth H.D. (2013). Hydrogels for controlled pulmonary delivery. Ther. Deliv..

[67] Selvam P., El-Sherbiny I.M., Smyth H.D. (2011). Swellable hydrogel particles for controlled release pulmonary administration using propellant-driven metered dose inhalers. J. Aerosol Med. Pulm. Drug Deliv..

[68] Bell C.L., Peppas N.A. (1996). Water, solute and protein diffusion in physiologically responsive hydrogels of poly(methacrylic acid-g-ethylene glycol). Biomaterials.

[69] Chaturvedi K., Ganguly K., Nadagouda M.N., Aminabhavi T.M. (2013). Polymeric hydrogels for oral insulin delivery. J. Control Release.

[70] Iwakura A., Fujita M., Kataoka K., Tambara K., Sakakibara Y., Komeda M., Tabata Y. (2003). Intramyocardial sustained delivery of basic fibroblast growth factor improves angiogenesis and ventricular function in a rat infarct model. Heart Vessels.

[71] Liu Y., Sun L., Huan Y., Zhao H., Deng J. (2006). Effects of basic fibroblast growth factor microspheres on angiogenesis in ischemic myocardium and cardiac function: Analysis with dobutamine cardiovascular magnetic resonance tagging. Eur. J. Cardiothorac. Surg..

[72] Headen D.M., Aubry G., Lu H., Garcia A.J. (2014). Microfluidic-based generation of size-controlled, biofunctionalized synthetic polymer microgels for cell encapsulation. Adv. Mater..

[73] Mao A.S., Shin J.W., Utech S., Wang H., Uzun O., Li W., Cooper M., Hu Y., Zhang L., Weitz D.A. (2017). Deterministic encapsulation of single cells in thin tunable microgels for niche modelling and therapeutic delivery. Nat. Mater..

[74] Annamalai R.T., Hong X., Schott N.G., Tiruchinapally G., Levi B., Stegemann J.P. (2019). Injectable osteogenic microtissues containing mesenchymal stromal cells conformally fill and repair critical-size defects. Biomaterials.

[75] Zhang S., Hu P., Liu T., Li Z., Huang Y., Liao J., Hamid M.R., Wen L., Wang T., Mo C. (2019). Kartogenin hydrolysis product 4-aminobiphenyl distributes to cartilage and mediates cartilage regeneration. Theranostics.

[76] Yang J., Zhu Y., Wang F., Deng L., Xu X., Cui W. (2020). Microfluidic liposomes-anchored microgels as extended delivery platform for treatment of osteoarthritis. Chem. Eng. J..

[77] Nisini R., Poerio N., Mariotti S., De Santis F., Fraziano M. (2018). The Multirole of Liposomes in Therapy and Prevention of Infectious Diseases. Front. Immunol..

[78] Bose S., Volpatti L.R., Thiono D., Yesilyurt V., McGladrigan C., Tang Y., Facklam A., Wang A., Jhunjhunwala S., Veiseh O. (2020). A retrievable implant for the long-term encapsulation and survival of therapeutic xenogeneic cells. Nat. Biomed. Eng..

[79] Morais A.I., Wang X., Vieira E.G., Viana B.C., Silva-Filho E.C., Osajima J.A., Afewerki S., Corat M.A., Silva H.S., Marciano F.R. (2020). Electrospraying Oxygen-Generating Microparticles for Tissue Engineering Applications. Int. J. Nanomed..

[80] Groll J., Boland T., Blunk T., Burdick J.A., Cho D.-W., Dalton P.D., Derby B., Forgacs G., Li Q., Mironov V.A. (2016). Biofabrication: Reappraising the definition of an evolving field. Biofabrication.

[81] Moroni L., Burdick J.A., Highley C., Lee S.J., Morimoto Y., Takeuchi S., Yoo J.J. (2018). Biofabrication strategies for 3D in vitro models and regenerative medicine. Nat. Rev. Mater..

[82] Murphy S.V., Atala A. (2014). 3D bioprinting of tissues and organs. Nat. Biotechnol..

[83] Kamperman T., Henke S., van den Berg A., Shin S.R., Tamayol A., Khademhosseini A., Karperien M., Leijten J. (2017). Single Cell Microgel Based Modular Bioinks for Uncoupled Cellular Micro- and Macroenvironments. Adv. Healthc. Mater..

[84] Xin S., Chimene D., Garza J.E., Gaharwar A.K., Alge D.L. (2019). Clickable PEG hydrogel microspheres as building blocks for 3D bioprinting. Biomater. Sci..

[85] Highley C.B., Song K.H., Daly A.C., Burdick J.A. (2019). Jammed Microgel Inks for 3D Printing Applications. Adv. Sci..

[86] O’Bryan C.S., Bhattacharjee T., Marshall S.L., Gregory Sawyer W., Angelini T.E. (2018). Commercially available microgels for 3D bioprinting. Bioprinting.

[87] Subbiah R., Hipfinger C., Tahayeri A., Athirasala A., Horsophonphong S., Thrivikraman G., França C.M., Cunha D.A., Mansoorifar A., Zahariev A. (2020). 3D Printing of Microgel-Loaded Modular Microcages as Instructive Scaffolds for Tissue Engineering. Adv. Mater..

[88] Zhang Y.S., Khademhosseini A. (2017). Advances in engineering hydrogels. Science.

[89] Samuel R., Duda D.G., Fukumura D., Jain R.K. (2015). Vascular diseases await translation of blood vessels engineered from stem cells. Sci. Transl. Med..

[90] Wimmer R.A., Leopoldi A., Aichinger M., Wick N., Hantusch B., Novatchkova M., Taubenschmid J., Hämmerle M., Esk C., Bagley J.A. (2019). Human blood vessel organoids as a model of diabetic vasculopathy. Nature.

[91] Zhao H., Wang Z., Jiang S., Wang J., Hu Z., Lobie P.E., Ma S. (2020). Microfluidic Synthesis of Injectable Angiogenic Microgels. Cell Rep. Phys. Sci..

[92] Jeon O., Lee Y.B., Jeong H., Lee S.J., Wells D., Alsberg E. (2019). Individual cell-only bioink and photocurable supporting medium for 3D printing and generation of engineered tissues with complex geometries. Mater. Horiz..

[93] Takei T., Nakahara H., Ijima H., Kawakami K. (2012). Synthesis of a chitosan derivative soluble at neutral pH and gellable by freeze-thawing, and its application in wound care. Acta Biomater..

[94] Saito T., Tabata Y. (2012). Preparation of gelatin hydrogels incorporating low-molecular-weight heparin for anti-fibrotic therapy. Acta Biomater..

[95] Abasalizadeh F., Moghaddam S.V., Alizadeh E., Akbari E., Kashani E., Fazljou S.M.B., Torbati M., Akbarzadeh A. (2020). Alginate-based hydrogels as drug delivery vehicles in cancer treatment and their applications in wound dressing and 3D bioprinting. J. Biol. Eng..

[96] Rice C.D., Dykstra M.A., Feil P.H. (1992). Microbial contamination in two antimicrobial and four control brands of alginate impression material. J. Prosthet. Dent..

[97] McCloskey A.P., Gilmore B.F., Laverty G. (2014). Evolution of antimicrobial peptides to self-assembled peptides for biomaterial applications. Pathogens.

[98] Mishra A., Loo Y., Deng R., Chuah Y.J., Hee H.T., Ying J.Y., Hauser C.A.E. (2011). Ultrasmall natural peptides self-assemble to strong temperature-resistant helical fibers in scaffolds suitable for tissue engineering. Nano Today.

[99] Loo Y., Wong Y.-C., Cai E.Z., Ang C.-H., Raju A., Lakshmanan A., Koh A.G., Zhou H.J., Lim T.-C., Moochhala S.M. (2014). Ultrashort peptide nanofibrous hydrogels for the acceleration of healing of burn wounds. Biomaterials.

[100] Hauser C.A., Zhang S. (2010). Designer self-assembling peptide nanofiber biological materials. Chem. Soc. Rev..

[101] Loo Y., Zhang S., Hauser C.A. (2012). From short peptides to nanofibers to macromolecular assemblies in biomedicine. Biotechnol. Adv..

[102] Loo Y., Chan Y.S., Szczerbinska I., Tan B.C.P., Wan A.C.A., Ng H.H., Hauser C.A.E. (2019). A Chemically Well-Defined, Self-Assembling 3D Substrate for Long-Term Culture of Human Pluripotent Stem Cells. ACS Appl. Bio. Mater..

[103] Arab W.T., Niyas A.M., Seferji K., Susapto H.H., Hauser C.A.E. (2018). Evaluation of peptide nanogels for accelerated wound healing in normal micropigs. J. Nanosci. Nanotechnol..

[104] Loo Y., Lakshmanan A., Ni M., Toh L.L., Wang S., Hauser C.A.E. (2015). Peptide Bioink: Self-Assembling Nanofibrous Scaffolds for Three-Dimensional Organotypic Cultures. Nano Lett..

[105] Seow W.Y., Kandasamy K., Purnamawati K., Sun W., Hauser C.A.E. (2019). Thin peptide hydrogel membranes suitable as scaffolds for engineering layered biostructures. Acta Biomater..

[106] Tian Y.F., Devgun J.M., Collier J.H. (2011). Fibrillized peptide microgels for cell encapsulation and 3D cell culture. Soft Matter.

[107] Tsuda Y., Morimoto Y., Takeuchi S. (2010). Monodisperse cell-encapsulating peptide microgel beads for 3D cell culture. Langmuir.

[108] Calderon G., Susapto H.H., Hauser C.A.E. Delivery of endothelial cell-laden microgel elicits angiogenesis in self-assembling ultrashort peptides.

[109] Ghalayini S., Susapto H.H., Hall S., Kahin K., Hauser C.A.E. (2019). Preparation and printability of ultrashort self-assembling peptide nanoparticles. Int. J. Bioprint..

[110] Khan Z., Kahin K., Rauf S., Ramirez-Calderon G., Papagiannis N., Abdulmajid M., Hauser C.A.E. (2019). Optimization of a 3D bioprinting process using ultrashort peptide bioinks. Int. J. Bioprint..

[111] Arab W., Kahin K., Khan Z., Hauser C.A.E. (2019). Exploring nanofibrous self-assembling peptide hydrogels using mouse myoblast cells for three-dimensional bioprinting and tissue engineering applications. Int. J. Bioprint..

[112] Reithofer M.R., Chan K.-H., Lakshmanan A., Lam D.H., Mishra A., Gopalan B., Joshi M., Wang S., Hauser C.A.E. (2014). Ligation of anti-cancer drugs to self-assembling ultrashort peptides by click chemistry for localized therapy. Chem. Sci..

[113] Wang F., Su H., Xu D., Dai W., Zhang W., Wang Z., Anderson C.F., Zheng M., Oh R., Wan F. (2020). Tumour sensitization via the extended intratumoural release of a STING agonist and camptothecin from a self-assembled hydrogel. Nat. Biomed. Eng..

